# Socioeconomic inequalities in COVID-19 infection and vaccine uptake among children and adolescents in Catalonia, Spain: a population-based cohort study

**DOI:** 10.3389/fped.2024.1466884

**Published:** 2024-11-19

**Authors:** Irene López-Sánchez, Aida Perramon-Malavez, Antoni Soriano-Arandes, Clara Prats, Talita Duarte-Salles, Berta Raventós, Elena Roel

**Affiliations:** ^1^ Real World Epidemiology Research Group, Fundació Institut Universitari per a la Recerca a l’Atenció Primària de Salut Jordi Gol I Gurina (IDIAPJGol), Barcelona, Spain; ^2^ Department of Medicine and Life Sciences, Universitat Pompeu Fabra, Barcelona, Spain; ^3^ Department of Physics, Universitat Politècnica de Catalunya (UPC-BarcelonaTech), Barcelona, Spain; ^4^ Paediatric Infectious Diseases and Immunodeficiencies Unit, Children’s Hospital, Vall d’Hebron Barcelona Hospital Campus, Barcelona, Spain; ^5^ Infection and Immunity in Paediatric Patients, Vall d’Hebron Research Institute, Barcelona, Spain; ^6^ Department of Medical Informatics, Erasmus University Medical Center, Rotterdam, Netherlands; ^7^ Department of Paediatrics, Obstetrics, Gynaecology and Preventive Medicine and Public Health, Universitat Autònoma de Barcelona, Bellaterra (Cerdanyola del Vallès), Barcelona, Spain; ^8^ Agència de Salut Pública de Barcelona, Barcelona, Spain

**Keywords:** COVID-19, vaccine uptake, infection, children, adolescents, socioeconomic deprivation

## Abstract

**Introduction:**

This study aims to investigate the relationship between deprivation, as measured by a socioeconomic deprivation index (SDI) score for census tract urban areas, and COVID-19 infections and vaccine uptake among children and adolescents before and after the vaccination rollout in Catalonia, Spain.

**Methods:**

We conducted a population-based cohort study using primary care records. Individuals were followed 3 months before the start of the vaccination campaign in Spain and 3 months after. Children (5–11 years) and adolescents (12–15 years) with at least 1 year of prior history observation available and without missing deprivation data. For each outcome, we estimated cumulative incidence and crude Cox proportional-hazard models by SDI quintiles, and hazard ratios (HRs) of COVID-19 infection and vaccine uptake relative to the least deprived quintile, Q1.

**Results:**

Before COVID-19 vaccination rollout, 290,625 children and 179,685 adolescents were analyzed. Increased HR of deprivation was associated with a higher risk of COVID-19 infection in both children [Q5: 1.55 (95% CI, 1.47–1.63)] and adolescents [Q5: 1.36 (95% CI, 1.29–1.43)]. After the rollout, this pattern changed among children, with lower risk of infection in more deprived areas [Q5: 0.62 (95% CI, 0.61–0.64)]. Vaccine uptake was higher among adolescents than children, but in both age groups, non-vaccination was more common among those living in more deprived areas (39.3% and 74.6% in Q1 vs. 26.5% and 66.9% in Q5 among children and adolescents, respectively).

**Conclusions:**

Children and adolescents living in deprived areas were at higher risk of COVID-19 non-vaccination. Socioeconomic disparities in COVID-19 infection were also evident before vaccine rollout, with a higher infection risk in deprived areas across age groups. Our findings suggest that changes in the association between deprivation and infections among children after the vaccine rollout were likely due to testing disparities.

## 1 Introduction

The coronavirus disease 2019 (COVID-19) pandemic disproportionately affected vulnerable populations with high socioeconomic deprivation (1). Although extensive research has already been conducted on this topic (2–7), the relationship between deprivation and COVID-19 infection and vaccination among children and adolescents remains unclear.

Research from the United Kingdom (UK) suggests that children and adolescents with high deprivation face a higher risk of low COVID-19 vaccine uptake compared to their less deprived peers (8). Conversely, studies from Canada and New Zealand seem to find vaccination rates negatively associated with deprivation (9–12). Additionally, research from the United States (US) show that children from low-income households are more susceptible to COVID-19 infection (13, 14). However, generalizing these findings to other regions may not be applicable, and evidence on whether vaccination campaigns have impacted this association is scarce.

In Spain, a prior study found a pattern of increased risk of infection with deprivation, which decreased after vaccine rollout (7). However, this evidence was limited to adults aged ≥40 years. Identifying socioeconomic disparities in COVID-19 vaccination and infection in younger populations could provide key information to guide immunization strategies in countries where vaccines are readily accessible. In this study, we aimed to investigate the association between deprivation and COVID-19 infection 3 months before the COVID-19 vaccine rollout and 3 months after the start of vaccination of children and adolescents living in urban areas of Catalonia, Spain. We also investigated the associations between deprivation and COVID-19 vaccine uptake among this population.

## 2 Materials and methods

### 2.1 Study design and data source

We conducted a population-based cohort study using primary care data from the Information System for Research in Primary Care (SIDIAP; www.sidiap.org) database, which contains pseudo-anonymized electronic health records of approximately 75% of the Catalan population and is representative in geography, age, and sex (15). This database has been standardized to the Observational Medical Outcomes Partnership (OMOP) Common Data Model (CDM), and the provenance of the data, specifically for capturing COVID-19 vaccination and infection has been well documented (16).

### 2.2 Study periods

**Figure 1:**
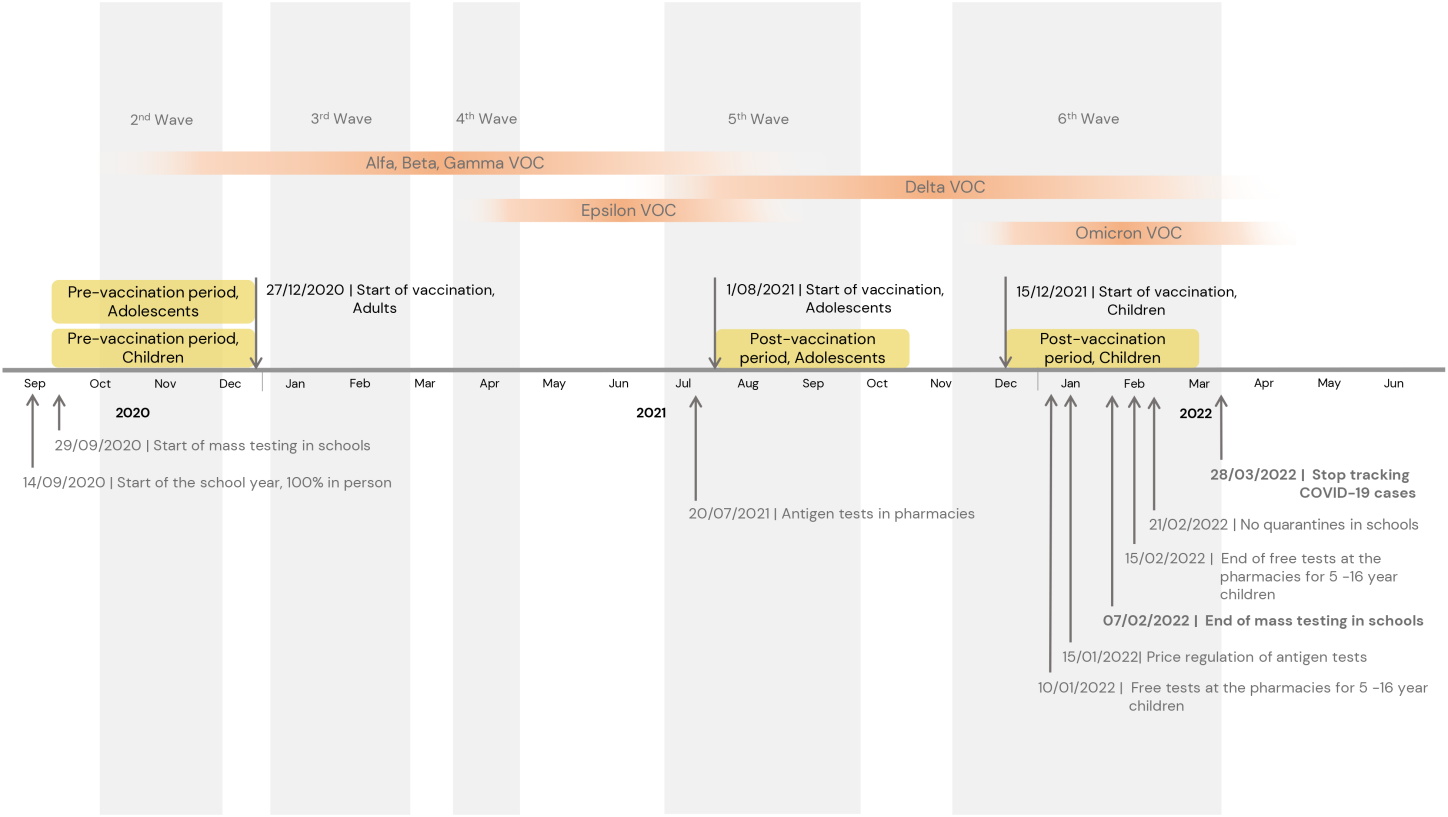
Timeline of the cohort studies. VOC, variant of concern; COVID-19; coronavirus disease 2019.

We followed children aged 5–11 years and adolescents aged 12–15 years during two time periods (1) a pre-vaccination period, three months before the start of the vaccination rollout in Spain, from the 26th of September to the 26th of December 2020 (17); and (2) a vaccination period, three months after the start of the vaccination rollout among adolescents [from the 1st August to the 1st November 2021 (18)] and children [from the 15th of December 2021 to the 15th of March 2022 (19)]. Thus, four cohorts were followed, one for each age group and time period (Figure 1), with the follow-up of all cohorts starting on the first day of each time period (index date). The vaccination period for children was set to three months, and, to ensure consistency across cohorts, the same time window was applied to the other cohorts (20, 21).

### 2.3 Study participants

**Figure 2:**
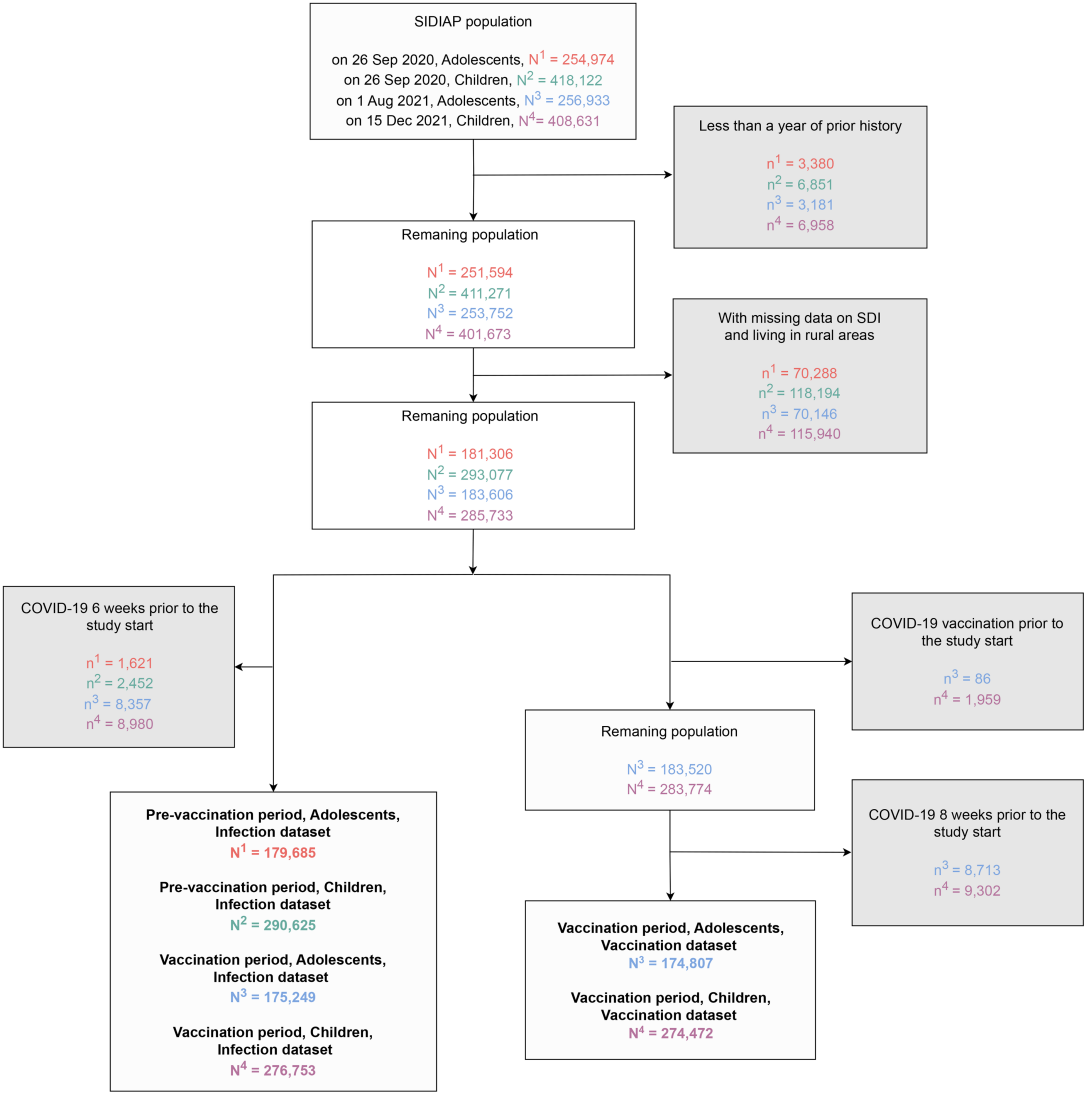
Flowchart showing the inclusion and exclusion criteria for study population in analysis of socioeconomic inequalities in COVID-19 vaccination and infection in children and adolescents, Catalonia, Spain. SDI, socioeconomic deprivation index; SIDIAP, information system for research in primary care; COVID-19; coronavirus disease 2019.

We included all children and adolescents registered in the SIDIAP database as of 26 September 2020 (index date for all participants). We excluded those with less than 1 year of prior medical history available at the index date and those with missing data on deprivation or living in rural areas (defined as municipalities with <10,000 inhabitants and a population density <150 habitants/km2) as this information was unavailable in these areas (Figure 2).

When analyzing COVID-19 infection, we excluded those individuals with an infection ≤6 weeks before the start of the study period. When analyzing COVID-19 vaccine uptake, we excluded those vaccinated before the vaccination period, and those who tested positive for COVID-19 within the previous 8 weeks, since Spanish policies required 8-week delay between infection and vaccination (22). Additionally, participants who had been infected before vaccination were censored at the date of infection due to infections being considered a competing risk for vaccination (23–25). Figure 2 shows the flow chart of inclusion and exclusion criteria.

For each period, children and adolescents were followed until the occurrence of the outcome of interest, end of the study period, exit from the SIDIAP database, or death, whichever occurred first.

### 2.4 Study setting

Our study was conducted against the backdrop of the evolving COVID-19 pandemic in Catalonia, Spain. The region experienced distinct phases of the pandemic, each marked by unique challenges and responses.

During the pre-vaccination period, Catalonia was experiencing the second wave of the COVID-19 pandemic, with incidence rates ranging from 120 to over 400 cases per 100,000 inhabitants (26, 27). In September 2020, educational settings reopened for in-person activities after closing in March 2020 and enforced stringent non-pharmaceutical interventions to mitigate the virus transmission. These measures consisted of handwashing, mandatory mask use for children over 5 years old, social distancing, organization of children and teachers into bubble groups to maintain the same groups of individuals and therefore facilitate contact tracing, and the improvement of ventilation of classrooms. Other public health measures such as mass testing were implemented in schools located in areas with high incidence of COVID-19 to reduce transmission (28, 29). In the event of a confirmed case, testing of all classmates was performed and, regardless of the PCR test result, the whole group was quarantined for 10 days (30).

In late July 2021, pharmacies began selling antigen tests following a surge of COVID-19 cases that coincided with the emergence of the Delta variant (31, 32). When the academic year 2021–2022 started in September after a two-month break, school protocols were revised. In the event of a confirmed infection, close contacts were required to undergo testing, which was provided free of charge through the public health system. Those who had previously been infected within the last 6 months or were fully vaccinated, were directed to obtain a supervised antigen test at authorized pharmacies. Individuals who did not meet these criteria were instructed to undergo a PCR test at a primary healthcare center (33). Between October and November 2021, COVID-19 incidence rates decreased from 1,630 to 716 cases per 100,000 inhabitants (26).

In the vaccination period for children, the incidence of COVID-19 in Catalonia increased sharply due to the higher transmissibility rate of the Omicron variant, rising from 551 cases in December 2021 to 3,285 cases per 100,000 inhabitants in January 2022, but then rapidly decreased to 300 cases per 100,000 inhabitants by mid-March when most of the population had been infected (26, 32). In January 2022, the government regulated the price of antigen tests to improve accessibility and offered free antigen tests to fully vaccinated children and adolescents when a positive case appeared in their social bubble (34, 35). Testing was restricted to unvaccinated children and adolescents without prior history of infection over the last three months. This measure ended in February 2022, along with mass testing and quarantines in schools (36–38).

### 2.5 Variables

Deprivation was assessed using a Socioeconomic Deprivation Index (SDI) score based on the place of residence, the *Mortalidad en áreas pequeñas españolas y desigualdades socioeconómicas y ambientales* deprivation index (39). This index was calculated at census tract areas using data from the 2001 national census in Spain, and was only available for urban areas (municipalities with >10,000 inhabitants and a population density >150 habitants/km2). This index has been linked to each individual's most recent residential address and categorized into five quintiles, the first being the least deprived (Q1).

Our outcomes of interest were COVID-19 infection and vaccination. COVID-19 infections were defined as a positive SARS-CoV-2 test result [antigen or polymerase chain reaction] or a clinical COVID-19 diagnosis without a record of a COVID-19 infection 6 weeks prior, with the test or diagnosis date as the date of infection (40). In each study period, we only considered the first COVID-19 infection per person. COVID-19 vaccination was defined as having received at least one dose of a COVID-19 vaccine. The date of vaccination was the date of the first dose administration.

### 2.6 Statistical analysis

We described participants’ characteristics at baseline and compared them to people with missing data on SDI using standardized mean differences (SMD). We calculated the cumulative incidence of COVID-19 infection by study period and age group, according to SDI quintile, by dividing the number of incident COVID-19 infections by the number of individuals at risk. Vaccination uptake was calculated at the end of the vaccination period by dividing the number of individuals vaccinated by the number of individuals eligible for vaccination. We also described the proportion of individuals who had undergone one or more tests registered in the public health system during follow-up, according to the deprivation quintile at the end of each study period.

To assess the association between SDI and COVID-19 infection, we performed crude Cox proportional-hazard regression models to estimate hazard ratios (HRs) with 95% confidence intervals (CI) of COVID-19 infection by SDI quintile relative to the least deprived quintile, Q1. To assess the association between SDI and COVID-19 vaccine uptake, we performed crude cause-specific hazard models to estimate the HR with 95% CIs of vaccination by SDI quintile relative to Q1 (41). All the analyses were stratified by age group and study period. We visually inspected log-log survival curves to check the proportional hazard assumptions for SDI (Supplementary Figure S1). We performed crude models based on our assumptions on the relationship between our exposure and our outcomes, which we represented with direct acyclic graphs (Supplementary Figures S2, S3). As a secondary analysis, we stratified our analyses by sex.

As a sensitivity analysis, we excluded individuals with any prior history of COVID-19 infection before the start of each study period, as it might have influenced the risks of infection as well as of being vaccinated.

We used R version 4.1 for data curation and analysis.

## 3 Results

Before the vaccination rollout 290,625 children [149,605 (51.5%) male] and 179,685 adolescents [92,442 (51.4%) male], were included (Tables 1, 2). Most participants were Spanish (over 85%), and the distribution of SDI quintiles was similar across age groups. Baseline characteristics were similar after the vaccination rollout, however, prior COVID-19 infection was low (0.6%) among children and adolescents in the pre-vaccination period, but increased to 17% in the vaccination period in both age groups (Tables 1, 2). Individuals excluded because of missing data on SDI showed minor differences (<0.2 SMD) (42) in baseline characteristics compared to those with complete data (Supplementary Table S1).

**Table 1:** Baseline characteristics of children included in the study of socioeconomic inequalities in COVID-19 infection and vaccination, Catalonia, Spain.*

Characteristics	Pre-vaccination period, infection	Vaccination period, infection	Vaccination period, vaccine uptake
*N*	290,625	276,753	274,472
Age [median (IQR)]	8.0 [6.0, 10.0]	8.0 [6.0, 10.0]	8.0 [6.0, 10.0]
Sex			
Female	141,020 (48.5)	134,520 (48.6)	133,468 (48.6)
Male	149,605 (51.5)	142,233 (51.4)	141,004 (51.4)
Nationality			
Spain	250,644 (86.2)	234,209 (84.6)	232,036 (84.5)
Africa	12,915 (4.4)	13,797 (5.0)	13,773 (5.0)
Asia & Oceania	9,492 (3.3)	9,441 (3.4)	9,430 (3.4)
Central & South America	8,802 (3.0)	10,577 (3.8)	10,544 (3.8)
Eastern Europe	5,487 (1.9)	5,251 (1.9)	5,225 (1.9)
Europe & North America	3,285 (1.1)	3,478 (1.3)	3,464 (1.3)
SDI quintile			
Q1 (least deprived)	53,140 (18.3)	50,188 (18.1)	49,699 (18.1)
Q2	55,703 (19.2)	52,301 (18.9)	51,795 (18.9)
Q3	56,850 (19.6)	53,707 (19.4)	53,234 (19.4)
Q4	60,099 (20.7)	57,426 (20.7)	56,973 (20.8)
Q5 (most deprived)	64,833 (22.3)	63,131 (22.8)	62,771 (22.9)
Previous COVID-19 Infection	1,814 (0.6)	47,300 (17.1)	46,640 (17.0)

* Values are no. (%) except as indicated. Pre-vaccination period (Sep 26–Dec 26 2020), Vaccination period, children (15 Dec 2021–15 Mar 2022). Quintiles listed from least deprived (Q1) to most deprived (Q5). IQR, interquartile range; SDI, socioeconomic deprivation index; COVID-19; coronavirus disease 2019.

**Table 2:** Baseline characteristics of adolescents included in the study of socioeconomic inequalities in COVID-19 infection and vaccination, Catalonia, Spain.*

Characteristics	Pre-vaccination period, infection	Vaccination period, infection	Vaccination period, vaccine uptake
*N*	179,685	175,249	174,807
Age [median (IQR)]	13.0 [12.0, 14.0]	13.0 [12.0, 14.0]	13.0 [12.0, 14.0]
Sex			
Female	87,243 (48.6)	84,727 (48.3)	84,516 (48.3)
Male	92,442 (51.4)	90,522 (51.7)	90,291 (51.7)
Nationality			
Spain	159,824 (88.9)	153,932 (87.8)	153,552 (87.8)
Africa	5,247 (2.9)	5,641 (3.2)	5,623 (3.2)
Asia & Oceania	5,065 (2.8)	5,329 (3.0)	5,316 (3.0)
Central & South America	4,908 (2.7)	5,508 (3.1)	5,490 (3.1)
Eastern Europe	3,030 (1.7)	3,182 (1.8)	3,174 (1.8)
Europe & North America	1,611 (0.9)	1,657 (0.9)	1,652 (0.9)
SDI quintile			
Q1 (least deprived)	34,524 (19.2)	32,518 (18.6)	32,440 (18.6)
Q2	35,637 (19.8)	34,477 (19.7)	34,397 (19.7)
Q3	35,686 (19.9)	34,788 (19.9)	34,700 (19.9)
Q4	35,994 (20.0)	35,595 (20.3)	35,495 (20.3)
Q5 (most deprived)	37,844 (21.1)	37,871 (21.6)	37,775 (21.6)
Previous COVID-19 Infection	1,039 (0.6)	30,323 (17.3)	29,958 (17.1)

* Values are no. (%) except as indicated. Pre-vaccination period (Sep 26–Dec 26 2020), vaccination period, adolescents (Aug 1–Oct 1 2021). Quintiles listed from least deprived (Q1) to most deprived (Q5). IQR, interquartile range; SDI, socioeconomic deprivation index; COVID-19; coronavirus disease 2019.

### 3.1 COVID-19 tests, cumulative incidence of infection and vaccine uptake

**Figure 3:**
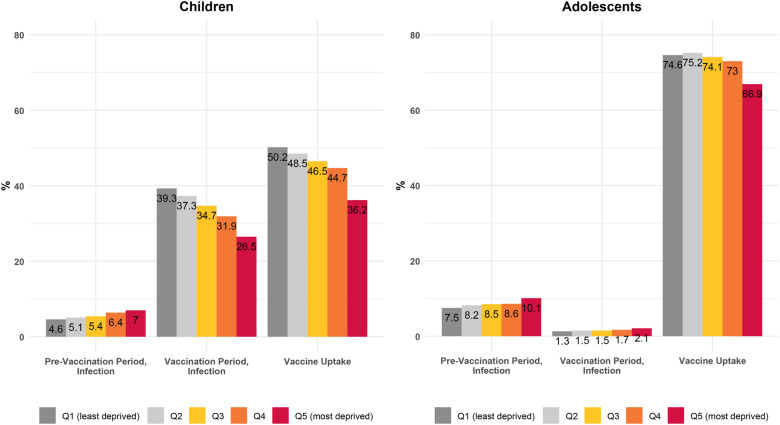
Cumulative incidence of COVID-19 infection and vaccine uptake by age group and socioeconomic deprivation index quintile, before and after the start of the vaccination rollout. Quintiles listed from least deprived (Q1) to most deprived (Q5).Pre-vaccination period (Sep 26–Dec 26 2020), vaccination period, adolescents (Aug 1–Oct 1 2021), Vaccination period, children (15 Dec 2021–15 Mar 2022). SDI, socioeconomic deprivation index.

Before vaccination rollout, children and adolescents living in more deprived areas were more COVID-19 tested than those living in less deprived areas: 32.1% (Q1), vs. 37.6% (Q5) in children and 45.9% (Q1), vs. 48.6% (Q5) in adolescents (Supplementary Figure S4). Cumulative incidence of COVID-19 infection was 5.8% in children and 8.6% in adolescents. Infections were more frequent in more deprived areas in both age groups, showing a clear gradient of increased infection with deprivation: 4.6% (Q1), 5.4% (Q3) and 7% (Q5) in children, and 7.5% (Q1), 8.5% (Q3) and 10.1% (Q5) in adolescents (Figure 3 and Supplementary Table S2).

After vaccination rollout, testing was higher among those living in less deprived areas: 71.4% (Q1) vs. 59.5% (Q5) in children and 17.9% (Q1), vs. 14.2% (Q5) in adolescents (Supplementary Figure S4). Cumulative incidence of infection was 33.6% for children and 1.6% for adolescents. The pattern of increased COVID-19 infections with increased deprivation was still seen among adolescents (1.3% [Q1], 1.5% [Q3] and 2.1% [Q5]). However, we found the opposite pattern among children, with those in the least deprived quintile having a higher incidence proportion of infection (39.3% Q1, 34.7% Q3 and 26.5% Q5).

Vaccination uptake was 44.8% for children and 72.6% for adolescents. We also found a pattern of increased vaccination among those living in less deprived areas: 51.9% (Q1) vs. 38.3% (Q5) in children and 84.8% (Q1) vs. 75.7% (Q5) in adolescents.

### 3.2 Association between SDI and COVID-19 infection and vaccine uptake

**Figure 4:**
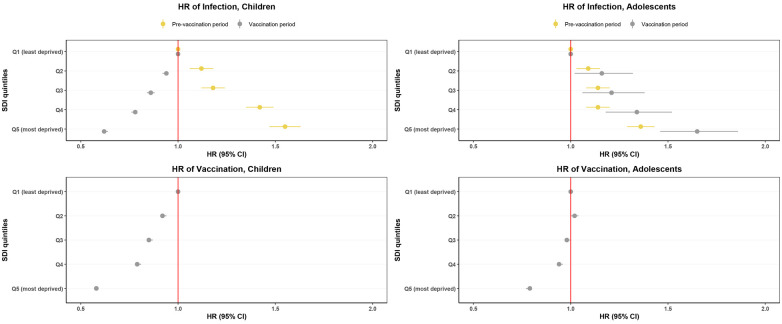
Hazard ratios of COVID-19 infection and vaccination 3 months before and 3 months after the start of the COVID-19 vaccination rollout by socioeconomic deprivation index quintile. Quintiles listed from least deprived (Q1) to most deprived (Q5). Pre-vaccination period (Sep 26–Dec 26 2020), vaccination period, adolescents (Aug 1–Oct 1 2021), vaccination period, children (15 Dec 2021–15 Mar 2022). Bars indicate 95% CI, dots indicate HR. SDI, socioeconomic deprivation Index. HR, hazard ratio.

Before the vaccination rollout, we observed a pattern of increased risks of COVID-19 infection with increased deprivation in both age groups (Figure 4 and Supplementary Table S3). For instance, among children, HR of infection relative to Q1 ranged from 1.12 (95% CI 1.06–1.18) in Q2 to 1.55 (1.47–1.63) in Q5 areas.

After vaccination rollout, COVID-19 infection risk was higher among adolescents living in more deprived areas, while in children, we observed an inverse pattern, with a lower risk of infection with more deprivation (Figure 4 and Supplementary Table S3). The HR of infection ranged from 1.16 (1.02–1.32) in Q2 to 1.65 (1.46–1.86) in Q5 areas for adolescents, and from 0.94 (0.92–0.95) in Q2 to 0.62 (0.61–0.64) in Q5 areas for children.

In both age groups, those living in more deprived areas had a lower risk of vaccination (Figure 4 and Supplementary Table S3). The HR ranged from 0.92 (0.91–0.94) in Q2 to 0.58 (0.57–0.59) in Q5 areas for children and from 1.02 (1.00–1.04) in Q2 to 0.79 (0.77–0.80) in Q5 areas for adolescents.

In our secondary analyses stratifying by sex, results were similar among both sexes. Results were also consistent after excluding individuals with a previous COVID-19 infection (Supplementary Table S3).

## 4 Discussion

In this population-based cohort study including 290,625 children and 179,685 adolescents, we observed a gradient of increased COVID-19 infection rates with more deprivation in children and adolescents prior to the vaccination rollout. However, following the vaccination rollout, this pattern reversed among children, with a higher proportion of infection in less deprived areas. Vaccination rates also differed by deprivation, with a gradient of increased vaccination with less deprivation among children and adolescents.

Several factors can explain the association between deprivation and recorded COVID-19 infections. Previous studies on infectious diseases, including COVID-19, have shown a positive association between disease incidence and deprivation, especially among adults (43–50). Residents of deprived areas, characterized by lower education levels, face increased exposure to COVID-19 due to factors such as low-occupation jobs with more precarious conditions, overcrowded households, and lower health literacy, which hinders their engagement with public health measures (51–53). While these conditions primarily affect adults, a similar trend has been observed among children, particularly within the most vulnerable populations. In the United States, ethnic minorities and socioeconomically disadvantaged children have borne a disproportionate share of COVID-19 infections (13, 14). This phenomenon may be partially attributable to intrafamily transmission dynamics (54). Indeed, the presence of overcrowded living conditions may be a contributing factor, as they are more prevalent in economically deprived regions and have previously been associated with an elevated risk of COVID-19-related mortality (55). It is worth noting that during the pre-vaccination period, intensive surveillance policies were implemented in schools, regardless of deprivation levels, which enabled a more accurate and equitable reflection of COVID-19 infections.

During the vaccination period in children, we observed higher COVID-19 infections in less socioeconomically deprived areas, possibly linked to evolving testing practices (Figure 1). During the vaccination period, schools changed their protocols and stopped conducting on-site tests. Free tests were made available at pharmacies, and children who had been in close contact with a positive case were advised to take a test. Although testing was readily accessible during this period, families were responsible for getting tested (Supplementary Figure S3) (56). The higher incidence of recorded COVID-19 cases among children in less deprived areas correlated with their higher testing rates. The increased rates of infection compared to adolescents and pre-vaccination period can be largely explained by the higher transmissibility of the Omicron variant that emerged during this period (57).

COVID-19 vaccine uptake disparities among children could be related to several factors, including household income, neighborhood, parental education, and vaccination status (Supplementary Figure S3) (58–61). In the UK, children aged 5–11 from the most deprived quintiles had lower first-dose vaccine uptake rates than their less deprived peers (6.6% vs. 15.6%), as did those aged 12–15 (38.3% vs. 68.3%) (8). Roel et al. found similar results when looking at the adult population aged ≥40 years in Catalonia, with higher odds of non-vaccination among persons living in more deprived areas (OR of 1.01 in Q2, and 1.33 in Q5) (7). While in our study there is a larger inequality in vaccination rates among children and adolescents compared to working-age groups, both studies highlight the significant impact of area deprivation on vaccination rates across different age groups. Factors such as infection patterns, vaccine prioritization strategies, educational level, and parental decision-making influenced by attitudes and beliefs about vaccines may have contributed to these disparities (58, 62). Overall, our results emphasize the need to address vaccine access barriers across all ages and socioeconomic levels.

The main strength of this study is that our results are representative of the pediatric population aged 5–15 years old residing in urban areas of Catalonia, providing a comprehensive understanding of COVID-19 infection and vaccine uptake in this region of Spain. In addition, the risk of misclassification bias for vaccination is low, since SIDIAP captures all vaccines administered in Catalonia and all COVID-19 tests performed within the public healthcare system.

This study also has limitations. First, we lacked information on individual-level deprivation, and therefore there is a risk of ecological bias. Secondly, deprivation was estimated based on data from the 2001 census national tract, whereas our study period spanned from 2020 to 2022. Additionally, deprivation was only available in urban areas, and therefore, results are not generalisable to rural populations. Thirdly, herd immunity, changes in testing patterns throughout the pandemic, and circulating COVID-19 variants may have affected the comparability of study periods in terms of COVID-19 infections. It is worth noting that the vaccination period partially coincided with school breaks for both children and adolescents. Although COVID-19 testing was widely available, holidays could have potentially affected the detection of COVID-19 cases, as testing efforts in schools were not in place during this time (29). Lastly, we were unable to control for potential confounding variables related to the parents.

In this study, we found a pattern of increased risk of COVID-19 non-vaccination with deprivation among children and adolescents. Infection inequalities persisted 3 months after vaccine rollout in adolescents. Possibly due to testing disparities, the situation reversed for children. These findings highlight the importance of addressing testing gaps, emphasizing the need for targeted vaccinations considering the socioeconomic context.

## Data Availability

The datasets presented in this article are not readily available because in accordance with current European and national law, the data used in this study is only available for the researchers participating in this study. Thus, we are not allowed to distribute or make publicly available the data to other parties. However, researchers from public institutions can request data from SIDIAP if they comply with certain requirements. Requests to access the datasets should be directed to https://www.sidiap.org/index.php/menu-solicitudesen/application-proccedure.
