# Supplementary material for: Socioeconomic inequalities in COVID-19 infection and vaccine uptake among children and adolescents in Catalonia, Spain: a population-based cohort study
Source: Front Pediatr. 2024 Nov 19;12:1466884. doi: 10.3389/fped.2024.1466884 (PMC11615722; doi:10.3389/fped.2024.1466884)
Supplement: Supplementary file 1 [file Datasheet1.pdf]

Supplementary Material

1 Supplementary Tables

Table S1. Baseline characteristics of study participants with and without missing data on Socioeconomic Deprivation Index

	**Pre-Vaccination Period, Children**			**Pre-Vaccination Period, Adolescents**			**Vaccination Period, Children**			**Vaccination Period, Adolescents**		
	With missing data	Without missing data		With missing data	Without missing data		With missing data	Without missing data		With missing data	Without missing data	
**Characteristic**	(column %)	(column %)	SMD	(column %)	(column %)	SMD	(column %)	(column %)	SMD	(column %)	(column %)	SMD
**N**	53,902	293,077		28,527	181,306		55,950	285,733		28,231	183,606	
**Age (median [IQR])**	8.0 [6.0, 10.0]	8.0 [6.0, 10.0]	0.116	13.0 [12.0, 14.0]	13.0 [12.0, 14.0]	<0.001	8.0 [6.0, 10.0]	8.0 [6.0, 10.0]	0.156	13.0 [12.0, 14.0]	13.0 [12.0, 14.0]	0.012
**Sex, male**	27,966 (51.9)	150,843 (51.5)	0.008	14,653 (51.4)	93,279 (51.4)	0.002	28,929 (51.7)	146,886 (51.4)	0.006	14,568 (51.6)	94,590 (51.5)	0.002
**Nationality**			0.155			0.131			0.177			0.129
	47,809 (88.7)	252,695 (86.2)		25,306 (88.7)	161,214 (88.9)		49,459 (88.4)	242,339 (84.8)		24,917 (88.3)	161,607 (88.0)	
	1,742 (3.2)	13,052 (4.5)		733 (2.6)	5,316 (2.9)		1,962 (3.5)	14,055 (4.9)		789 (2.8)	5,797 (3.2)	

	1,141<br>(2.1)	8,945<br>(3.1)		728<br>(2.6)	5,007<br>(2.8)		1,343<br>(2.4)	10,788<br>(3.8)		775<br>(2.7)	5,757<br>(3.1)	
	876<br>(1.6)	9,563<br>(3.3)		474<br>(1.7)	5,101<br>(2.8)		806<br>(1.4)	9,589<br>(3.4)		485<br>(1.7)	5,473<br>(3.0)	
	1,422<br>(2.6)	5,522<br>(1.9)		819<br>(2.9)	3,051<br>(1.7)		1,369<br>(2.4)	5,392<br>(1.9)		788<br>(2.8)	3,250<br>(1.8)	
	912<br>(1.7)	3,300<br>(1.1)		467<br>(1.6)	1,617<br>(0.9)		1,011<br>(1.8)	3,570<br>(1.2)		477<br>(1.7)	1,722<br>(0.9)	

IQR, interquartil range; SMD, standardized mean difference

Table S2. Cumulative incidence of COVID-19 infection and vaccine coverage by cohort, overall and by sex and Socioeconomic Deprivation Index quintile*

	Overall	Q1	Q2	Q3	Q4	Q5
**Pre-vaccination rollout, Children**						
Infection						
Overall	167,73 (5.8)	2,441 (4.6)	2,862 (5.1)	3,058 (5.4)	3,867 (6.4)	4,545 (7.0)
Female	8,122 (5.8)	1,174 (4.6)	1,366 (5.1)	1,488 (5.4)	1,912 (6.6)	2,182 (6.9)
Male	8,651 (5.8)	1,267 (4.6)	1,496 (5.2)	1,570 (5.4)	1,955 (6.3)	2,363 (7.1)
**Pre-vaccination rollout, Adolescents**						
Infection						
Overall	15,430 (8.6)	2,597 (7.5)	2,908 (8.2)	3,039 (8.5)	3,078 (8.6)	3,808 (10.1)
Female	7,542 (8.6)	1,261 (7.5)	1,406 (8.1)	1,464 (8.4)	1,529 (8.8)	1,882 (10.3)
Male	7,888 (8.5)	1,336 (7.6)	1,502 (8.2)	1,575 (8.6)	1,549 (8.4)	1,926 (9.8)
**Vaccination rollout, Children**						
Infection						
Overall	92,958 (33.6)	19,729 (39.3)	19,501 (37.3)	18,656 (34.7)	18,334 (31.9)	16,738 (26.5)
Female	44,677 (33.2)	94,36 (38.7)	9,414 (37.2)	9,079 (34.7)	8,697 (31.2)	8,051 (26.1)
Male	48,281 (33.9)	10,293 (39.9)	10,087 (37.3)	9,577 (34.8)	9,637 (32.6)	8,687 (26.9)
Vaccination						
Overall	12,3018 (44.8)	24,970 (50.2)	25,117 (48.5)	24,743 (46.5)	25,465 (44.7)	22,723 (36.2)
Female	59,975 (44.9)	12,141 (50.2)	12,173 (48.6)	11,998 (46.2)	12,484 (45.1)	11,179 (36.5)
Male	63,043 (44.7)	12,829 (50.2)	12,944 (48.4)	12,745 (46.7)	12,981 (44.3)	11,544 (35.9)
**Vaccination rollout, Adolescents**						
Infection						

Overall	2,815 (1.6)	408 (1.3)	502 (1.5)	527 (1.5)	598 (1.7)	780 (2.1)
Female	1,466 (1.7)	208 (1.3)	266 (1.6)	284 (1.7)	293 (1.7)	415 (2.3)
Male	1,349 (1.5)	200 (1.2)	236 (1.3)	243 (1.4)	305 (1.7)	365 (1.9)
Vaccination						
Overall	126,995 (72.6)	24,216 (74.6)	25,853 (75.2)	25,716 (74.1)	25,924 (73.0)	25,286 (66.9)
Female	61,712 (73.0)	11,662 (74.3)	12,540 (75.5)	12,533 (74.2)	12,724 (74.1)	12,253 (67.5)
Male	65,283 (72.3)	12,554 (75.0)	13,313 (74.9)	13,183 (74.0)	13,200 (72.0)	13,033 (66.4)

*Values are no. (%) except as indicated. Pre-vaccination period (Sep 26 - Dec 26 2020), Vaccination period, adolescents (Aug 1 - Oct 1 2021), Vaccination period, children (15 Dec 2021 - 15 Mar 2022). Quintiles listed from least deprived (Q1) to most deprived (Q5). IQR, interquartile range; SDI, Socioeconomic Deprivation Index.

Table S3. Crude Hazard Ratios of COVID-19 infection and vaccination 3 months before and after vaccination rollout by Socioeconomic Deprivation Index quintile by SDI quintile relative to Q1, stratified by sex and previous COVID-19 infection.

	**HR (95% CI)**	**HR Female (95% CI)**	**HR Male (95% CI)**	**HR Without Previous COVID-19 (95% CI)**
**Infection, Pre-vaccination period, Children**				
Q1 (ref.)	1 [Reference]	1 [Reference]	1 [Reference]	1 [Reference]
Q2	1.12 (1.06 - 1.18)	1.12 (1.04 - 1.21)	1.12 (1.04 - 1.21)	1.12 (1.06 - 1.18)
Q3	1.18 (1.12 - 1.24)	1.18 (1.10 - 1.28)	1.17 (1.09 - 1.26)	1.17 (1.11 - 1.24)
Q4	1.42 (1.35 - 1.49)	1.46 (1.35 - 1.57)	1.38 (1.28 - 1.48)	1.41 (1.34 - 1.49)
Q5	1.55 (1.47 - 1.63)	1.54 (1.43 - 1.65)	1.56 (1.46 - 1.67)	1.55 (1.47 - 1.63)
**Infection, Pre-vaccination period, Adolescents**				
Q1 (ref.)	1 [Reference]	1 [Reference]	1 [Reference]	1 [Reference]
Q2	1.09 (1.03 - 1.15)	1.08 (1.01 - 1.17)	1.09 (1.01 - 1.17)	1.09 (1.03 - 1.15)
Q3	1.14 (1.08 - 1.20)	1.13 (1.05 - 1.22)	1.15 (1.07 - 1.23)	1.13 (1.08 - 1.20)
Q4	1.14 (1.08 - 1.20)	1.18 (1.09 - 1.27)	1.11 (1.03 - 1.20)	1.14 (1.08 - 1.20)
Q5	1.36 (1.29 - 1.43)	1.40 (1.31 - 1.51)	1.32 (1.23 - 1.41)	1.36 (1.29 - 1.43)
**Infection, Vaccination period, Children**				
Q1 (ref.)	1 [Reference]	1 [Reference]	1 [Reference]	1 [Reference]
Q2	0.94 (0.92 - 0.95)	0.96 (0.93 - 0.98)	0.92 (0.89 - 0.94)	0.94 (0.92 - 0.96)
Q3	0.86 (0.84 - 0.88)	0.88 (0.85 - 0.90)	0.84 (0.82 - 0.87)	0.86 (0.84 - 0.87)
Q4	0.78 (0.76 - 0.79)	0.77 (0.75 - 0.79)	0.78 (0.76 - 0.80)	0.78 (0.76 - 0.79)
Q5	0.62 (0.61 - 0.64)	0.63 (0.61 - 0.65)	0.62 (0.60 - 0.64)	0.62 (0.61 - 0.64)
**Infection, Vaccination period, Adolescents**				
Q1 (ref.)	1 [Reference]	1 [Reference]	1 [Reference]	1 [Reference]
Q2	1.16 (1.02 - 1.32)	1.21 (1.01 - 1.45)	1.11 (0.92 - 1.34)	1.13 (0.98 - 1.30)
Q3	1.21 (1.06 - 1.38)	1.27 (1.06 - 1.52)	1.14 (0.95 - 1.38)	1.19 (1.03 - 1.36)
Q4	1.34 (1.18 - 1.52)	1.29 (1.08 - 1.54)	1.40 (1.17 - 1.67)	1.35 (1.18 - 1.55)
Q5	1.65 (1.46 - 1.86)	1.73 (1.47 - 2.05)	1.56 (1.31 - 1.86)	1.60 (1.41 - 1.82)
**Vaccination, Vaccination period, Children**				
Q1 (ref.)	1 [Reference]	1 [Reference]	1 [Reference]	1 [Reference]
Q2	0.92 (0.91 - 0.94)	0.93 (0.90 - 0.95)	0.92 (0.90 - 0.94)	0.92 (0.91 - 0.94)

Q3	0.85 (0.84 - 0.87)	0.85 (0.83 - 0.87)	0.86 (0.83 - 0.88)	0.85 (0.84 - 0.87)
Q4	0.79 (0.78-0.81)	0.80 (0.78 - 0.82)	0.78 (0.76 - 0.80)	0.79 (0.78-0.81)
Q5	0.58 (0.57 - 0.59)	0.59 (0.57 - 0.60)	0.58 (0.56 - 0.59)	0.57 (0.56 - 0.58)
**Vaccination, Vaccination period, Adolescents**				
Q1 (ref.)	1 [Reference]	1 [Reference]	1 [Reference]	1 [Reference]
Q2	1.02 (1.00 - 1.04)	1.03 (1.01 - 1.06)	1.01 (0.99 - 1.03)	1.02 (1.00 - 1.04)
Q3	0.98 (0.97 - 1.00)	0.99 (0.97 - 1.02)	0.98 (0.95 - 1.00)	0.98 (0.96 - 1.00)
Q4	0.94 (0.93 - 0.96)	0.98 (0.95 - 1.00)	0.91 (0.89 - 0.94)	0.94 (0.92 - 0.96)
Q5	0.79 (0.77 - 0.80)	0.81 (0.79 - 0.83)	0.77 (0.75 - 0.79)	0.77 (0.76 - 0.79)

Quintiles listed from least deprived (Q1) to most deprived (Q5). SDI, Socioeconomic Deprivation Index; COVID-19; Coronavirus Disease 2019. Pre-vaccination period (Sep 26 - Dec 26 2020), Vaccination period, adolescents (Aug 1 - Oct 1 2021), Vaccination period, children (15 Dec 2021 - 15 Mar 2022).

2 Supplementary Figures

Figure S1. Log-log survival curves to COVID-19 infection or vaccination by Socioeconomic Deprivation Index quintile, for each study period.

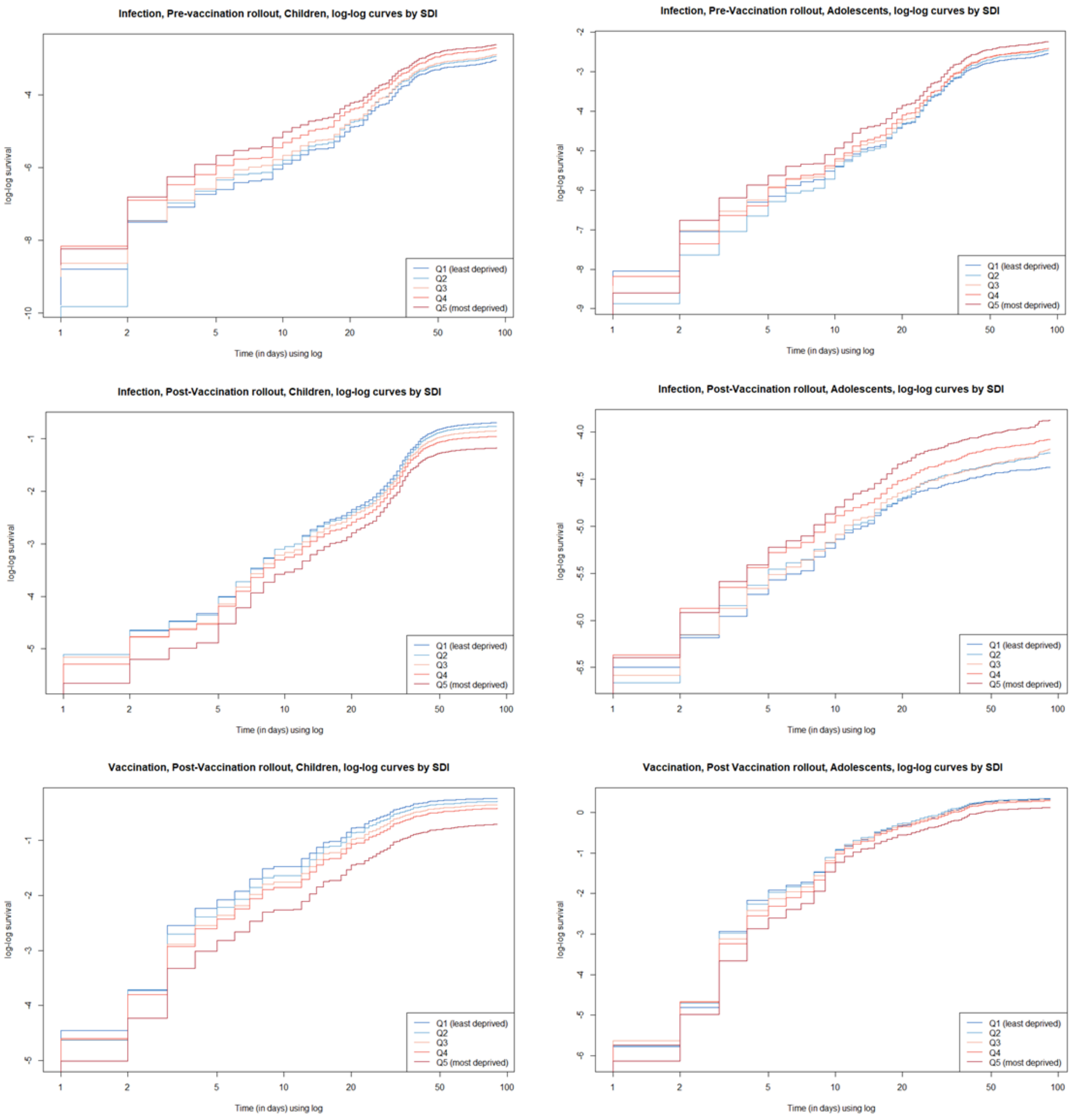

Pre-vaccination period (Sep 26 - Dec 26 2020), Vaccination period, adolescents (Aug 1 - Oct 1 2021), Vaccination period, children (15 Dec 2021 - 15 Mar 2022). Quintiles listed from least deprived (Q1) to most deprived (Q5). IQR, interquartile range; SDI, Socioeconomic Deprivation Index

Figure S2. Directed Acyclic Graph modelling the relationship between socioeconomic deprivation and COVID-19 infection among children and adolescents.

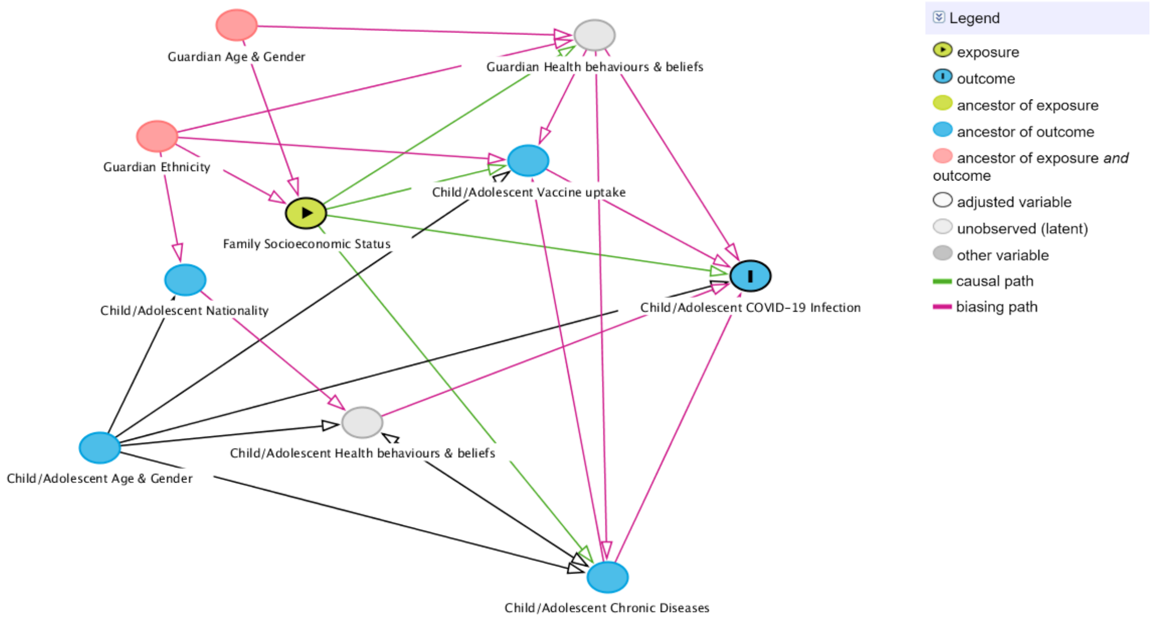

COVID-19; Coronavirus Disease 2019

Figure S3. Directed Acyclic Graph modelling the relationship between socioeconomic deprivation and COVID-19 vaccine uptake among children and adolescents.

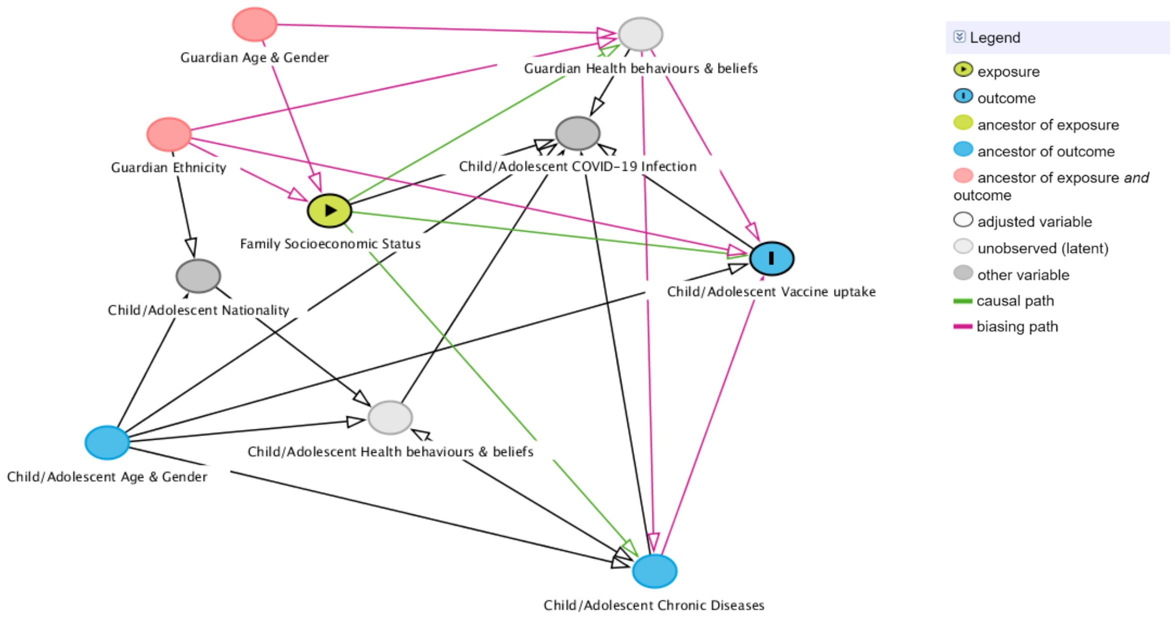

COVID-19; Coronavirus Disease 2019

Figure S4. Proportion of individuals who had performed one or more tests by age group and Socioeconomic Deprivation Index quintile, before and after the start of the vaccination rollout.

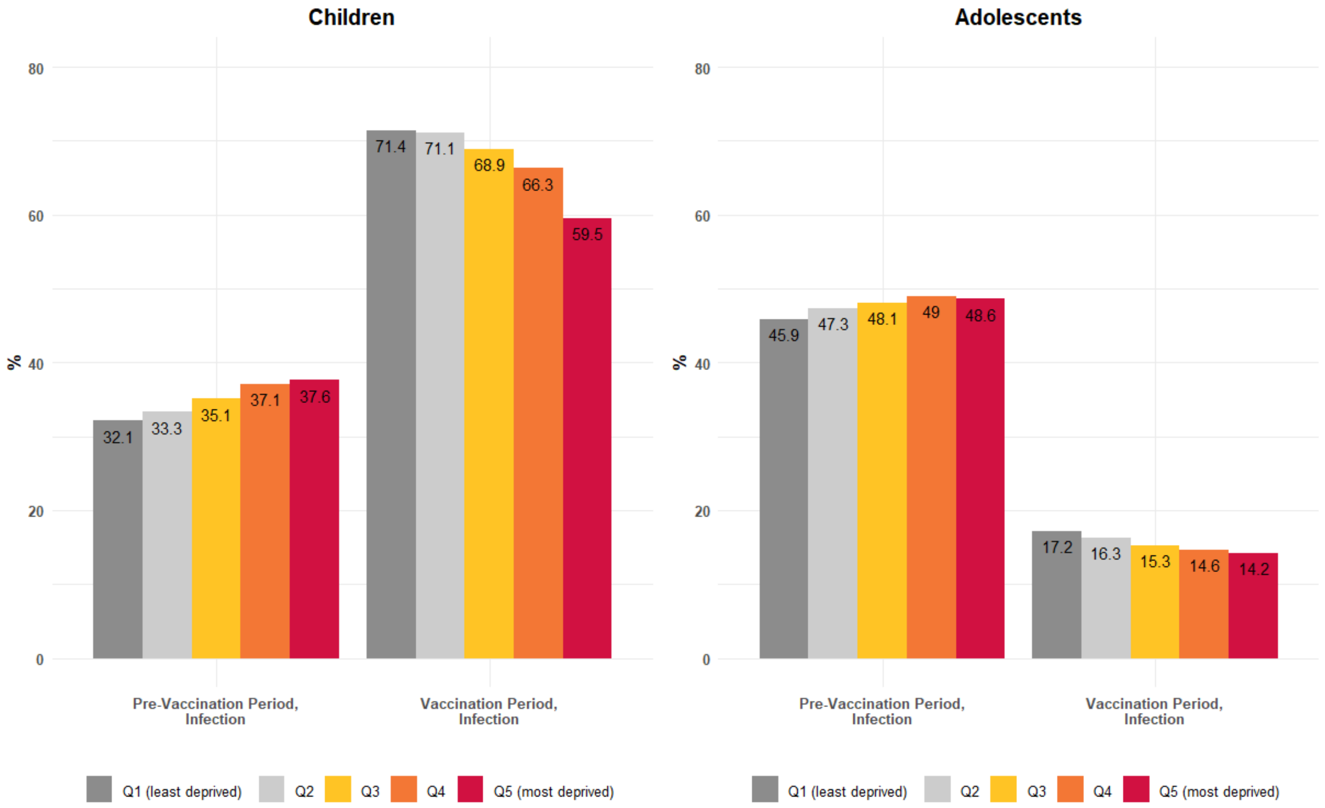

Quintiles listed from least deprived (Q1) to most deprived (Q5). SDI, Socioeconomic Deprivation Index. Pre-vaccination period (Sep 26 - Dec 26 2020), Vaccination period, adolescents (Aug 1 - Oct 1 2021), Vaccination period, children (15 Dec 2021 - 15 Mar 2022).
